# Molecular Confirmation of Massive *Taenia pisiformis* Cysticercosis in One Rabbit in Poland

**DOI:** 10.3390/pathogens10081029

**Published:** 2021-08-14

**Authors:** Małgorzata Samorek-Pieróg, Jacek Karamon, Adam Brzana, Ewa Bilska-Zając, Jolanta Zdybel, Tomasz Cencek

**Affiliations:** 1 Department of Parasitology and Invasive Diseases, National Veterinary Research Institute, Partyzantów Avenue 57, 24-100 Puławy, Poland; j.karamon@piwet.pulawy.pl (J.K.); ewa.bilska@piwet.pulawy.pl (E.B.-Z.); j.zdybel@piwet.pulawy.pl (J.Z.); tcencek@piwet.pulawy.pl (T.C.); 2 Regional Veterinary Laboratory, Wrocławska 170, 46-020 Opole, Poland; a.brzana@gmail.com

**Keywords:** *Taenia pisiformis*, rabbit, PCR, Poland

## Abstract

The aim of this study was to provide molecular characterization, together with phylogenetic analysis, of *Taenia pisiformis* cysts isolated from rabbit. On the basis of morphological features and molecular analysis, the cysticerci were identified as *T.* *pisiformis* metacestodes. PCR was performed with three different protocols to obtain partial sequences of 12S ribosomal RNA (12S rRNA), NADH dehydrogenase subunit 1 (nad1), and cytochrome oxidase subunit 1 (cox1) of *Taenia* spp. The products from the PCRs were sequenced. Interpretation of the sequencing results of the obtained amplicons, by comparing them with the GenBank database, proved that the causative agent, in this case, was *T. pisiformis*. The phylogenetic analysis of the received sequences identified a new haplotype. The received data can be used to supplement the species description. To our knowledge, this is the first molecular confirmation of *T. pisiformis* metacestodes infection in the rabbit, in Poland.

## 1. Introduction

*Taenia pisiformis* (Bloch, 1780) is a tapeworm with a worldwide geographical distribution. It belongs to the Taeniidae family and has an indirect life cycle. The final hosts are canids (rarely felids), and the intermediate hosts are lagomorphs (hares, rabbits) or rodents. The intermediate host becomes infected by eating eggs of the parasite excreted with the feces of canids. Swallowed eggs hatch in the small intestine of this host. Liberated oncospheres pass through the intestinal wall and the portal system to the liver. After 2–4 weeks, the juvenile stages cross the liver parenchyma to the abdominal cavity, where they develop into the metacestode stage (*cysticercus*) attached to the wall of the mesentery and omentum. The cysts are fluid-filled, with a globular to oval shape. The size of the cysts are 5–7 × 5–12 mm [1]. In the case of massive infections, they can fill the entire body cavity. The definitive host is infected by consuming the internal organs of an intermediate host infected with *T. pisiformis* metacestodes. In the small intestine of canids, the larval stages of the parasite develop into the adult worms, which mature after around 6–8 weeks and start producing eggs. The proglottids with eggs are released into the environment with the host′s excrements. Infection is usually asymptomatic in both the definitive and intermediate host. However, heavy infections can lead to liver damage in the intermediate host, and as a consequence hepatitis and cirrhosis can occur [1,2].

Although *T. pisiformis* is a tapeworm that occurs almost all over the globe [2], it is unclear whether it is a native or introduced species [3]. Infections with this parasite mainly affect wild hares; nevertheless, it may cause economic losses in the rabbit breeding industry worldwide. As *T. pisiformis* metacestodes rarely gives rise to specific symptoms, it is difficult to detect and is often diagnosed only during autopsy. In Poland, a study on slaughtered rabbits from small farms and industrial farms revealed the prevalence of this parasite to be 4.74% [4]. Cumulative research results from the 1990s show much more extensive *T. pisiformis* cysticercosis infections in hares from various regions of Poland (10.38%), while in large-scale rabbit farms, cysticercosis was recorded in about 5% of slaughtered rabbits [5]. In Mexico, the first formal report on infection with *T. pisiformis* metacestodes revealed a prevalence of about 70% [3]. Keith et al. [6], in their research in Canada, revealed the prevalence of *T. pisiformis* in the snowshoe hare population to have a mean level of 8%. *T. pisiformis* metacestodes in rabbits or hares have also been detected in many other places; for example, in Macronesia [7], Tenerife (Canary Islands) [8], Italy [9], and Yemen [10], proving its ubiquitous character.

Rabbits, as breeding animals, are characterized by high indexes of functional traits that determine their economic usefulness. For this reason, researchers often aim to eliminate diseases that contribute to economic losses on farms. In China, *T. pisiformis* cysticercosis is a common disease that affects rabbits, and because of the absence of effective vaccines and deworming drugs, this parasitic disease is not yet under control. The key to reducing the occurrence of this parasite seems to be in interrupting the transmission cycle between the intermediate host and the definitive host. Canids play a crucial role in transmission of *T. pisiformis*, therefore, investigations into the detection of this parasite in definitive hosts should be intensified [11]. The occurrence of *T. pisiformis* in definitive hosts has been described in most cases in connection with the detection of other parasites [12,13,14,15,16]. In Poland, the presence of this parasite in definitive hosts was detected by PCR during studies on *E. multilocularis* [17,18]. However, there was no confirmation of the species affiliation of larval forms in intermediate hosts. This prompted us to conduct the research described in this paper.

The purpose of this study was to present the molecular characterization, as well as a phylogenetic analysis, of *T. pisiformis* cysts isolated from rabbit.

## 2. Results

**Figure 1:**
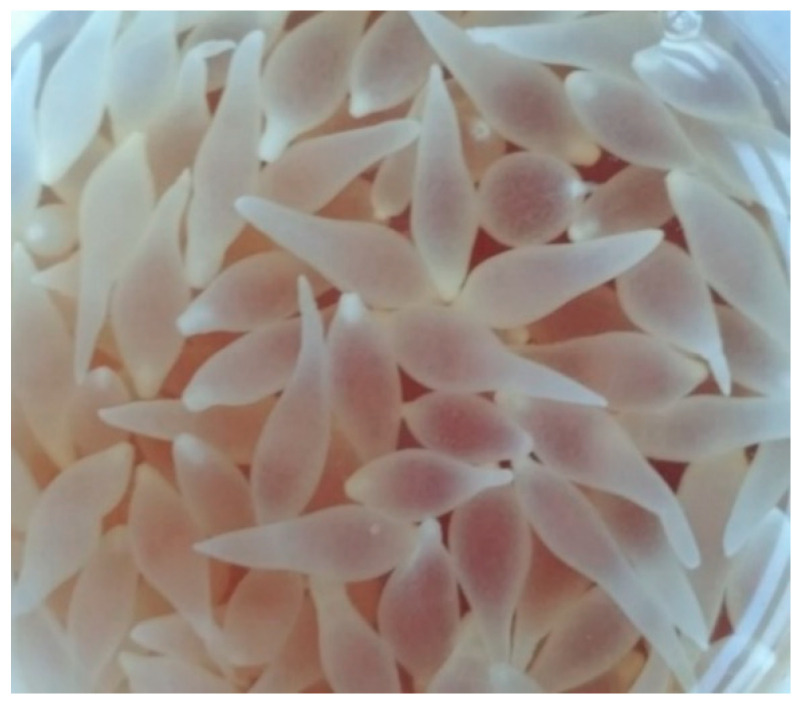
Cysts of *T. pisiformis* collected from the abdominal cavity of a rabbit.

**Figure 2:**
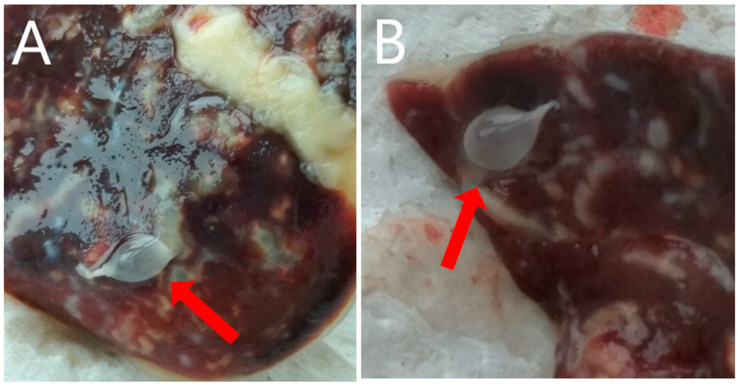
Cysticercosis caused by *T. pisiformis* on the liver surface (**A**,**B**) of the examined rabbit.

Post-mortem, macroscopic examination of the rabbit by an authorized veterinarian revealed highly developed ascites (fluid volume approx. 600 mL). In the fluid and within the liver (on its surface and in the flesh), numerous (248) small, pear-like, transparent cysts, with dimensions of 5–7 mm × 5–12 mm (Figure 1 and Figure 2) were present. The parasite was microscopically identified by measurement and shape, and compared to the description provided by Loos-Frank [1], which accurately matched the description of the metacestode stage of *T. pisiformis*. Moreover, the microscopic examination showed highly developed inflammatory, post-inflammatory, and degenerative changes of the liver associated with the presence of tapeworm cysts, and also catarrhal enteritis (mainly of the small intestine).

Amplification of partial sequences of 12S rRNA and the nad1 and cox1 genes was successful. The products of all PCR reactions were of the expected size. A comparison of the sequencing results of the obtained amplicons, using the GenBank database, confirmed that the detected parasite was *Taenia pisiformis*.

The contig of partial 12S rRNA forward and reverse sequences was 254 bp in length, which covers 34.9% (254 bp/728 bp) of the whole length. The alignment of our sequence with a sequence from Australia (accession number KJ591572.1) available from the GenBank database showed that there was 100% identity among them, confirming the species identification.

For further phylogenetic analysis, we used sequences of partial nad1 and cox1 genes, which were submitted to GenBank database under the accession numbers MZ287426 (partial cox1 gene) and MZ287427 (partial nad1 gene).

**Figure 3:**
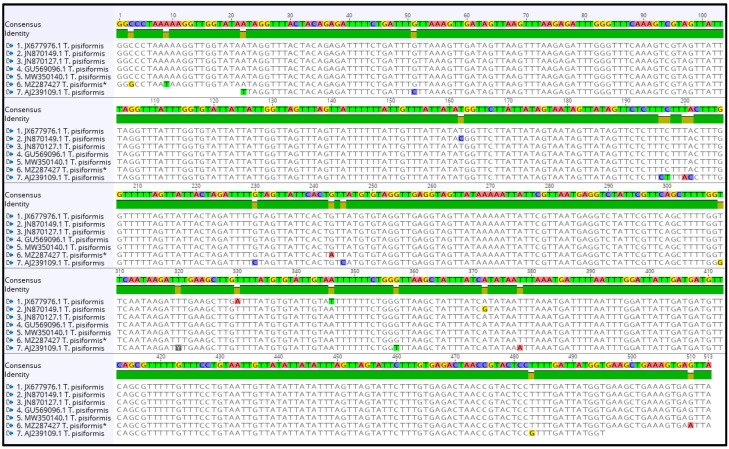
Alignment of the partial nad1 sequences of *T. pisiformis* available from the GenBank database (JN870127.1; JN870149.1; JX677976.1; MW350140.1; GU569096.1; AJ239109.1) and sequence MZ287427 T. pisiformis * (* denotes sequence from this study).

The contig of partial nad1 forward and reverse sequences was 513 bp in length, which covers 57.2% (513 bp/897 bp) of the whole length. The alignment of sequences of *T. pisiformis* available from the GenBank database (JN870127.1; JN870149.1; JX677976.1; MW350140.1; GU569096.1; AJ239109.1) with our sequence (MZ287427) showed that there was from 97.15% to 99.2% identity among them, differing from each other by single nucleotide variants (Figure 3). The G + C content was almost at the same level, in the range of 26.2% to 27.1%. The average base composition of the partial nad1 gene was 25% (A), 7.5% (C), 19.1% (G), and 48.3% (T). No insertions, deletions, or stop codons were observed. The comparison of the nad1 sequences revealed that all of the 21 nucleotide differences were attributable to substitutions, of which 5 (24%) were transversions and 16 (76%) were transitions.

**Figure 4:**
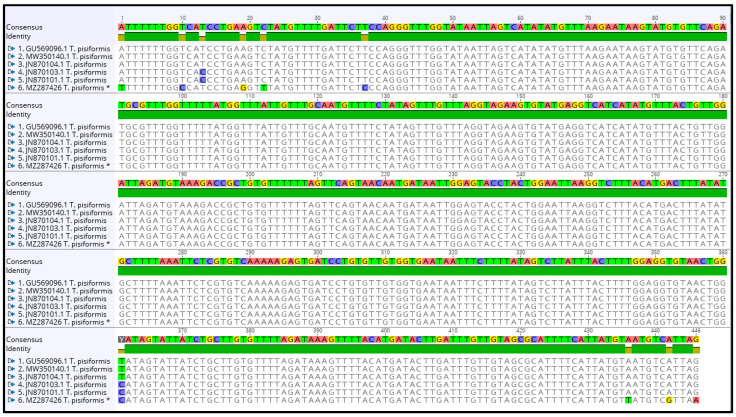
Alignment of the partial cox1 sequences of *T. pisiformis* available from the GenBank database (JN870103.1; JN870104.1; JN870101.1; MW350140.1; GU569096.1) with sequence MZ287426 T. pisiformis * (* denotes sequence from this study).

The contig of partial cox1 forward and reverse sequences was 446 bp in length, which covers 27.3% (446 bp/1620 bp) of the whole length. The alignment of sequences of *T. pisiformis* available from the GenBank database (JN870103.1; JN870104.1; JN870101.1; MW350140.1; GU569096.1) with our sequence (MZ287426) showed that there is 97.98% identity among them, differing from each other by single nucleotide variants (Figure 4). The G + C content was almost at the same level, in the range of 30.7% to 31.4%. The average base composition of the partial nad1 gene was 24.6% (A), 10.1% (C), 20.9% (G), and 44.5% (T). No insertions, deletions, or stop codons were observed. Comparison of the cox1 sequences revealed that all of the 10 nucleotide differences were attributable to substitutions, of which 3 (30%) were transversions and 7 (70%) were transitions.

**Figure 5:**
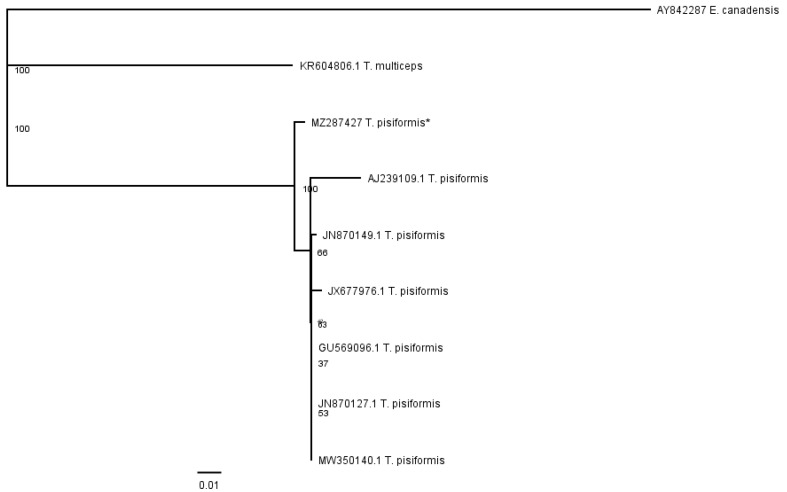
Phenogram of partial nad1 *T. pisiformis* sequences together with *T. multiceps* and *E. canadensis* as outgroups; * denotes sequence from this study.

The phenogram in Figure 5 was constructed from the partial nad1 alignment of our sequence (MZ287427) with sequences of *T. pisiformis* genotypes deposited in the GenBank (JN870127.1; JN870149.1; JX677976.1; MW350140.1; GU569096.1; AJ239109.1). The sequences of *T. multiceps* (KR604806.1) and *E. canadensis* (AY842287) were added as outgroups.

**Figure 6:**
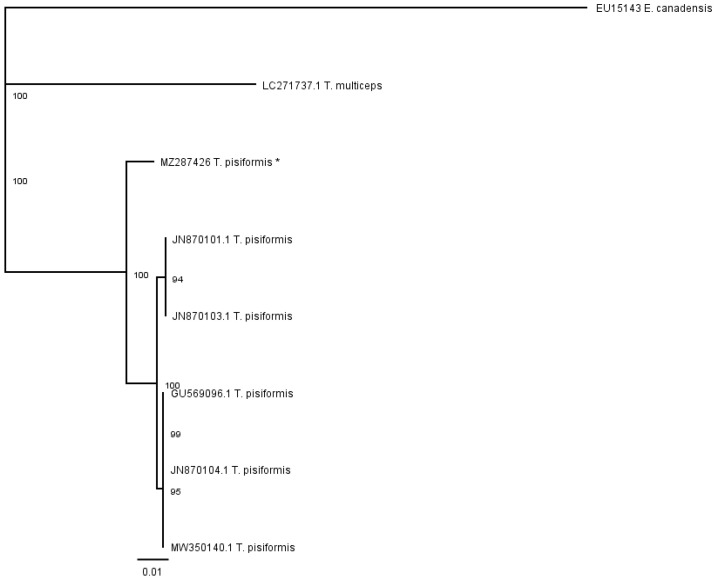
Phenogram of partial cox1 *T. pisiformis* sequences, together with *T. multiceps* and *E. canadensis* as outgroups; (* denotes sequence from this study).

The phenogram in Figure 6 was constructed from the partial cox1 alignment of our sequence (MZ287426) with sequences of *T. pisiformis* genotypes deposited in the GenBank (JN870103.1; JN870104.1; JN870101.1; MW350140.1; GU569096.1). The sequences of *T. multiceps* (LC271737.1) and *E. canadensis* (EU15143) were added as outgroups.

The genetic relationships of the analyzed species determined by the partial nad 1 sequence data were compared with those obtained using partial cox1 data. In both cases, the cladograms show that there were no significant subdivisions among the *T. pisiformis* sequences based on bootstrap support.

## 3. Discussion

Most of the research on *T. pisiformis* cysticercosis identification has been based on morphological characterization during autopsy. There is little information in the literature regarding molecular identification of this parasite. The GenBank database has a limited number of partial DNA sequences of cytochrome oxidase subunit 1 (cox1), NADH dehydrogenase subunit 1 (nad1), 12S ribosomal RNA, and the complete mitochondrion genome. To our knowledge, this is the first study providing molecular confirmation of *T. pisiformis* cysticercosis in an intermediate host, such as the rabbit, in Poland.

It is well known that *T. pisiformis* is a typical parasite of lagomorphs, especially rabbits and hares [3,4,5,6,7,8,9,10,19,20,21]. To date, this parasite has mostly been examined using the traditional detection method—identification of the macroscopic characteristics of the cysticercus and diagnosis by host specificity after autopsy. This is time consuming and laborious, and not always associated with accurate recognition. Moreover, cysticercosis is, in most cases, asymptomatic, and does not cause clinically significant symptoms. Nevertheless, Hallal-Calleros et al. [21] observed a loss of fertility in rabbits suffering from *T. pisiformis* cysticercosis, and Alzaga et al. [19] noted its negative relationship to kidney fat in the Iberian hare. Arias-Hernandez et al. [20] showed that rabbits infected with this parasite have modified metabolic characteristics (e.g., lipid metabolism), which may affect production and animal welfare. Moreover, in the case of obesity, these changes are intensified. Stancampiano et al. [9] concluded that infection by *T. pisiformis* in brown hares is significantly related to age, sampling year, and low full-weight. In published data, the relationship between the sex and age of the host and the presence of the parasites is not consistent. Some authors have noted the highest intensities of this parasites occur in juveniles [22], while others have recorded a peak of intensity in adult hares [9,19]. In terms of sex, some authors [3,23] have suggested that females are more susceptible to infection than males. In our study, the analyzed rabbit was taken away from its owner due to poor conditions and improper treatment, so it was impossible determine the factor that contributed most to the death of the animal—parasites or bad conditions. Nonetheless, through analyzing the microscopic examination described in this paper, we noticed similarities with the results of Stancampiano et al. [9], especially in hares with a high number of cysts on the surface of the liver. In their study, macroscopic examinations of individual positive animals were related to microscopic features of hepatic lesions, which means that the more cysticerci were found on the liver surface, the more alterations were discovered (e.g., inflammatory lympho-plasmatic foci within the liver parenchyma, chronic interstitial hepatitis (150); cysticerci, multifocal lymphocytic foci, multifocal to disseminated interstitial chronic hepatitis (202); cysticerci, scattered inflammatory lympho-plasmacystic foci within the liver parenchyma (180)). In our case, the number of cysticerci on the liver surface and in the fluid of the body cavity was 248, and alterations, such as highly developed inflammatory, post-inflammatory, and degenerative changes of the liver associated with the presence of tapeworm cysts, were present. Our results are also in line with other research groups, e.g., Alzaga et al. [19] and Allan et al. [24], who found a negative relationship between body condition and worm burden.

*T. pisiformis* is a tapeworm with a global distribution, thus mixed infections with other parasites are common. In order to make a precise diagnosis, there is a need to identify individual species from mixed infections of parasites, both in intermediate and definitive hosts. This is possible through the use of molecular methods which, once developed, can be easily applied to larvae, adult worms, eggs, and field samples. This may support precise and rapid identification [25]. Transmission of this parasite is sustained by the predator-prey relationship of the definitive and intermediate hosts. *T. pisiformis* infections often cause much more severe damage in intermediate hosts than in definitive hosts, where infections are mostly subclinical [26].

In our investigation, to confirm species affiliation, PCR for amplification of the 12S rRNA gene was performed. The received product was the expected size and, after sequencing, showed 100% identity with a sequence from Australia (accession number KJ591572.1). In Stancampiano et al.′s study [9], there was 99% similarity to the sequence from Japan (accession number AB329716).

In our further analysis, we focused on mitochondrial genes. They are one of the most popular molecular markers, and have been widely used in molecular ecology, population genetics, and diagnosis of parasitic organisms. Genes such as cox1 and nad1 are widely used as representative mitochondrial markers, because they are flanked by regions of sequence conservation, and “universal” PCR primer sets can be readily designed [27]. These genes can be used for determining intra-regional genetic characteristics that could be applied to studying the epidemiology and transmission of *T. pisiformis* [28].

In our study, the nucleotide compositions of the new sequences (in both examined partial genes) were biased toward A and T, with T being the most preferred nucleotide and C the last preferred; this is also consistent with other studies [27,29]. The same situation was found in the case of AT richness. The AT contents were 69% (cox1) and 73.4% (nad1); similarly, in Jia et al.′s [27] study, it was 73.2%, and in the study by Yang et al. [29] it was over 70%. It is worth noting that cox genes are characterized as having the lowest AT richness amongst *Taenia* species, in comparison to long and short non-coding regions [27].

The analysis of the sequence of the partial cox 1 gene revealed that it is closely related to samples from China (JN870103.1; JN870104.1; JN870101.1; MW350140.1; GU569096.1). The phylogenetic analysis produced a dendrogram with low bootstrap values, suggesting a lack of evolutionary differentiation among the *T. pisiformis* sequences. Our results were in line with Jia et al.′s [27] study, where it was also deduced that the cytochrome oxidase subunit 1 gene is amongst the slowest evolving and least variable of the mtDNA types. This may lead to the consideration of other molecular markers, especially in mixed infections.

The analysis of the sequence of the partial nad 1 gene revealed it is closely related to samples from China (JN870127.1; JN870149.1; JX677976.1; MW350140.1; GU569096.1) [27] and Australia (AJ239109.1) [30]. The alignment of sequences of *T. pisiformis* described in the results section showed that there is 97.15% to 99.2% identity among them, suggesting that the sequences were not genetically differentiated and had low levels of genetic diversity.

## 4. Materials and Methods

### 4.1. Case Description

The subject of the study was an adult crossbred female rabbit, approximately 2 years old. The animal came from an area located in the Opolskie Voivodeship, in southwestern Poland. The rabbit was taken away from the owner by the Society for the Protection of Animals due to poor housing conditions and treatment. Shortly thereafter, the animal died.

### 4.2. Autopsy and Sample Collection

Autopsy revealed numerous cysts on the surface of the liver, within the liver parenchyma, and in the fluid of the body cavity. The cysts were collected, placed in 70% ethanol, and sent to the laboratory of the National Veterinary Research Institute. The obtained material was frozen at –20 °C in individual tubes for further molecular analysis.

### 4.3. DNA Isolation and PCR Amplification

DNA was isolated according to manufacturer′s instructions from each individual cyst sample, using the QIAamp DNA Mini Kit (QIAGEN, Hilden, Germany).

PCR was performed with three different protocols. The first was a multiplex PCR by Trachsel et al. [31] with some minor modifications and was used to confirm preliminarily the species affiliation. It was carried out with three pairs of primers. The one pair (Cest3 and Cest5) amplified a fragment of 267 bp from 12S rRNA gene of *Taenia* spp. The two other pairs (Cest1 and Cest2; Cest4 and Cest5) amplified DNA from *Echinococcus multilocularis* and *Echinococcus granulosus*, respectively. The PCR amplification was performed using a commercial Multiplex PCR Kit (Qiagen, Hilden, Germany). The reaction mixture (50 µL) included 25 µL of Master Mix, 10 pmol of each primer, 15 µL nuclease-free water (Sigma-Aldrich, St. Louis, MO, USA), and 2 µL of DNA. The PCR was performed with an initial denaturation step at 95 °C for 15 min, followed by 40 cycles, with each cycle consisting of denaturation at 94 °C for 30 s, annealing at 58 °C for 90 s, elongation at 72 °C for 10 s, and a final extension step at 72 °C for 5 min.

The second and the third PCR protocols (for detection of partial mitochondrial genes) were conducted to obtain products for further phylogenetic analysis. The second PCR, by Bowles and McManus [32] and modified by Dybicz et al. [33], with some minor modifications, was carried out for the amplification of NADH dehydrogenase subunit 1 (nad1) using the primers JB11 (5′-AGATTCGTAAGGGGCCTAATA-3′) and JB12 (5′-ACCACTAACTAATTCACTTTC-3′), and amplified a fragment of about 500 bp. The PCR amplification was performed using a commercial Taq PCR Core Kit (Qiagen, Hilden, Germany). The reaction mixture (50 µL) included 5 µL of 10 × PCR Buffer, 10 pmol of each primer, 200 pmol of each dNTP, 2 µL of 25 mM MgCl_2_, 36.8 µL of nuclease-free water (Sigma-Aldrich, St. Louis, MO, USA), 1 U of Taq DNA Polymerase, and 1 µL of DNA. The PCR was performed with an initial denaturation step at 95 °C for 3 min, followed by 35 cycles, with each cycle consisting of denaturation at 95 °C for 60 s, annealing at 50 °C for 60 s, elongation at 72 °C for 60 s, and a final extension step at 72 °C for 5 min.

The third PCR was carried out for amplification of cytochrome oxidase subunit 1 (cox1) using the primers CO1F (5′-TTTTTTGGCCATCCTGAGGTTTAT-3′) and CO1R (5′-TAACGACATAACATAATGAAAATG-3′), as in Bowles et al. [34] and modified by Casulli et al. [35], with some minor modifications. This PCR amplified a fragment of 446 bp. The PCR amplification was performed using a commercial Taq PCR Core Kit (Qiagen, Hilden, Germany). The reaction mixture (50 µL) included 5 µL of 10 × PCR Buffer, 12.5 pmol of each primer, 200 pmol of each dNTP, 39.3 µL of nuclease-free water (Sigma-Aldrich, St. Louis, MO, USA), 1 U of Taq DNA Polymerase, and 1 µL of DNA. The PCR was performed with an initial denaturation step at 94 °C for 7 min, followed by 38 cycles, with each cycle consisting of denaturation at 94 °C for 30 s, annealing at 55 °C for 30 s, elongation at 72 °C for 30 s, and a final extension step at 72 °C for 5 min.

The sequences of mitochondrial partial nad1 and cox1 genes obtained from the last two PCR reactions, as popular genetic markers in genetics, were used for phylogenetic analysis. For each PCR reaction, a negative control (nuclease free water) and positive controls (template DNA from *Taenia hydatigena, Echinococcus granulosus* and *Echinococcus multilocularis*) were included. For the detection, 10 μL of the PCR products were electrophoresed on 2% agarose gels, with an added 5 μL of SimplySafe stain (EURx, Gdańsk, Poland). Electrophoresis was conducted using a Wide Mini-SubCell GT chamber and Power Pac Basic (Bio-Rad, Hercules, CA, USA). After electrophoresis, the gels were visualized using the Fusion Fx, Fusion Capt Advance software supplied by Vilber Lourmat (Collégien, France).

### 4.4. Sequencing and Phylogenetic Analysis

Positive products from the PCR reactions were sequenced. Sequencing was performed using a BigDye™ Terminator v3.1 Cycle Sequencing kit (Applied Biosystems, Foster City, CA, USA) on an ABI3730xl Genetic Analyzer (Applied Biosystems). The forward and reverse sequences were analyzed, aligned, and trimmed using the ClustalW algorithm in the Geneious R11 bioinformatics software platform. The consensus sequences were analyzed and compared to the GenBank Collection using the BLAST nucleotide algorithm to confirm the species identification. Sequences were submitted to the GenBank database under the accession numbers MZ287426 and MZ287427. Furthermore, phylogenetic analysis was conducted using sequences available in GenBank as outgroups. The phenogram was created applying the Tamura-Nei genetic distance model and Neighbour-Joining building method with 1000 bootstrap replications in the Geneious R11.

## 5. Conclusions

To conclude, this is the first molecular confirmation of *T. pisiformis* cysticercosis in rabbit, in Poland. Molecular methods of diagnosis enable accurate and precise diagnosis of the investigated parasite. What is more, knowledge about its genetic characteristics could be used to examine the epidemiology and transmission. In this study, partial sequences of the mitochondrial cytochrome oxidase subunit 1 (cox1) and NADH dehydrogenase subunit 1 (nad1) were analyzed. The phylogenetic analysis of the received sequences identified a new haplotype. The received data can be used to supplement the species description. The range of this infection among lagomorphs in Poland is still unknown, and further investigation is required. Our results provide useful knowledge for monitoring changes in parasite populations for future control strategies.

## Data Availability

The partial nucleotide sequences of *Taenia pisiformis* mitochondrial cytochrome oxidase subunit 1 (cox1) gene and NADH dehydrogenase subunit 1 (nad1) gene were deposited in the NCBI database and are publicity available under accession numbers MZ287426 and MZ287427, respectively.
